# Knowledge, Attitudes, and Intention to Report Child Abuse and Neglect, Including Dental Neglect, Among Dental Students in Plovdiv, Bulgaria: A Cross-Sectional Survey

**DOI:** 10.3390/healthcare14182942

**Published:** 2026-09-10

**Authors:** Martina Kancheva, Mariana Dimitrova-Haruil, Desislava Voynikova, Sevda Rimalovska

**Affiliations:** 1 Department of Pediatric Dentistry, Faculty of Dental Medicine, Plovdiv Medical University, 4000 Plovdiv, Bulgaria; mariana.dimitrova@mu-plovdiv.bg (M.D.-H.); sevda.rimalovska@mu-plovdiv.bg (S.R.); 2 Department of Algebra and Geometry, Faculty of Mathematics and Informatics, University of Plovdiv Paisii Hilendarski, 4000 Plovdiv, Bulgaria; desi_voynikova@uni-plovdiv.bg

**Keywords:** dental neglect, child abuse, neglect, education, knowledge

## Abstract

Background/Objectives: Dentists are uniquely positioned to detect child abuse and neglect (CAN), including dental neglect, yet reporting rates remain low and Bulgarian data are scarce. This study assessed the knowledge, attitudes, and self-reported intention to report CAN, including dental neglect, among clinical dental students in Plovdiv, Bulgaria, and their views on further education. We additionally tested whether recognition and attitudes differed by sex, stage of training, and prior CAN education, and identified independent correlates of the intention to report. Methods: A single-centre, analytical cross-sectional survey (Microsoft Forms) was conducted among 4th-, 5th-, and 6th-year (intern) dental students at the Medical University of Plovdiv (March–May 2025). The 17-item questionnaire covered demographics, knowledge, attitudes, the perceived role of the dentist, and interest in further training. An indicator-recognition score (0–24) was derived from four “select-all-that-apply” items. Group differences were examined with Mann–Whitney U and χ^2^/Fisher tests; independent correlates were identified with a general linear model (recognition score) and binary logistic regression (reporting intention; dentist’s role), with effect sizes reported. Results: Of 200 invited students, 115 responded (57.5%; 74 female, 41 male). Physical abuse was recognised by all participants (100%) but neglect by only ~75%. Female students scored higher than males (adjusted means 19.97 vs. 18.08; *p* = 0.005; partial η^2^ = 0.07), with no difference by year of study. Overall recognition scores were similar in students with and without prior CAN education (*p* = 0.713), but trained students more often endorsed the dentist’s safeguarding role (88.3% vs. 57.9%; *p* = 0.001) and intended to report suspected cases (66.2% vs. 39.5%; *p* = 0.011). In multivariable models, prior education (adjusted OR 3.31) and the recognition score (adjusted OR 1.32 per point) were each independently associated with the intention to report (both *p* ≤ 0.012). Over half (51%) considered their training insufficient and 94.8% wanted further education, preferring case-based seminars (71.3%). Conclusions: A clear gap exists between awareness of CAN and readiness to act; prior education related to readiness to act rather than to broader recognition. Structured, practice-oriented training with clear local reporting pathways and interprofessional learning is needed.

## 1. Introduction

According to the World Health Organization, child maltreatment is defined as “the abuse and neglect that occurs to children under 18 years of age. It includes all types of physical and/or emotional ill-treatment, sexual abuse, neglect, negligence and commercial or other exploitation, which results in actual or potential harm to the child’s health, survival, development or dignity” [1].

In Bulgaria, the National Program for the Prevention of Violence and Abuse of Children (2023–2026) reports that the Child Protection Departments at the Social Assistance Directorates received 1387 reports of violence in 2021. These departments are legally mandated to take protective measures and provide support to children at risk and their families [2].

One of the most persistent misconceptions about child abuse is the belief that it always involves physical violence. In reality, many children suffer significant harm through neglect, or through inaction by parents, guardians, or society at large. Neglect is defined as “the failure of a parent, guardian, or caregiver to ensure the development of the child in one of the following areas: health, education, emotional development, nutrition, provision of a home, and safety, when they are in a position to do so” [2,3].

Neglect may manifest in different forms, including physical, emotional, educational, and medical neglect. Some classifications further distinguish dental neglect as a separate subtype. The American Academy of Pediatric Dentistry defines dental neglect as “the willful failure of a parent or guardian, despite having adequate access to dental care, to seek and follow through with treatment necessary to ensure a level of oral health essential for adequate function and freedom from pain and infection” [4].

Dental neglect may include both acts of omission (e.g., not treating dental caries) and commission (e.g., refusing preventive care), and can be an indicator of broader patterns of child neglect [4,5,6,7]. This positions dental professionals uniquely to identify early signs of child abuse and neglect. Despite this, the rate of reporting by dentists remains low [8].

In dental education, the topic of child abuse and neglect (CAN) is typically addressed in courses such as Pediatric Dentistry, Forensic Medicine, and Public Health. However, research has shown that knowledge alone does not always translate into appropriate reporting behaviors [9,10,11,12,13]. In some countries, failure to report suspected abuse is a legal offense punishable by imprisonment [14]. Within Europe, the medicolegal framework for reporting child physical abuse by oral health professionals, including data-protection obligations under the General Data Protection Regulation had recently been characterized in a Portuguese cohort [15]. Although such penalties are not currently enforced in Bulgaria, this underscores the importance of comprehensive education and preparedness to act in the best interest of pediatric patients.

Internationally, studies among health-professional students and practitioners consistently reveal gaps between awareness of CAN and the confidence to act. Unsatisfactory knowledge, limited recognition of neglect, and barriers such as fear of negative consequences and uncertainty about reporting have been described among medical and nursing students in Greece [16] and dental professionals in Lithuania [17], with comparable shortfalls among dental students and graduates in Saudi Arabia, Germany, Turkey, Brazil, and North Macedonia [10,13,18,19,20]. This suggests that limited preparedness is a cross-cultural rather than a purely local challenge, and establishing where Bulgarian dental students stand within this picture is an important step toward targeted curricular reform.

To our knowledge, there is limited data on how well dental students in Bulgaria are educated and prepared to recognise and respond to CAN, including dental neglect. The primary aim of this study was to assess the knowledge, attitudes, and self-reported intention to report child abuse and neglect, including dental neglect, among clinical dental students (4th, 5th, and 6th year) at the Faculty of Dental Medicine in Plovdiv, Bulgaria, and to evaluate their views on the need for further education. Because the study relied on a self-administered questionnaire, it assessed self-reported knowledge, attitudes, and intention rather than clinically demonstrated competence or actual reporting behaviour.

Beyond describing these distributions, we sought to identify factors associated with students’ recognition of maltreatment indicators and with their self-reported intention to report. We specified the following hypotheses: (H1) indicator-recognition scores differ according to sex and stage of clinical training; and (H2) students who had received prior education on CAN demonstrate higher recognition scores and more favourable attitudes toward the dentist’s safeguarding role and the reporting of suspected cases than students without such education. We further examined, in multivariable models, whether prior education and recognition level were independently associated with the intention to report.

## 2. Materials and Methods

### 2.1. Study Design and Setting

This was a single-centre, analytical cross-sectional survey conducted at the Faculty of Dental Medicine, Medical University of Plovdiv, Bulgaria, between March and May 2025. The study was designed and reported in accordance with the STROBE (Strengthening the Reporting of Observational Studies in Epidemiology) statement for cross-sectional studies.

### 2.2. Participants and Recruitment

The target population comprised dental medicine students in the clinical phase of their training, defined as those enrolled in the 4th, 5th, and 6th (intern) years during the study period. All eligible students were invited to take part using a convenience sampling approach; participation was voluntary and anonymous. Fourth- and fifth-year students were recruited in person during scheduled lectures by means of a QR code linking to the questionnaire, while sixth-year interns, who no longer attend scheduled lectures, were invited by institutional e-mail. Of 200 students invited, 115 returned a completed questionnaire, yielding a response rate of 57.5%. Because all questionnaire items were set as mandatory, no partially completed responses were obtained and there were no missing data at the item level.

### 2.3. Ethical Considerations

The study was conducted in accordance with the principles of the Declaration of Helsinki. Ethical approval was obtained from the Research Ethics Committee of the Medical University of Plovdiv (approval No. P-KHE-23/31.12.2024). Before accessing the questionnaire, all participants were informed of the study’s purpose, the voluntary and anonymous nature of participation, and the intended use of the data; written informed consent was obtained from each participant prior to enrolment. No personally identifying information was collected.

### 2.4. Questionnaire

Data were collected using a structured, self-administered online questionnaire created in Microsoft Forms (Microsoft Corp., Redmond, WA, USA). The questionnaire items were developed on the basis of previously published instruments and literature on child abuse and neglect (CAN) in dental settings [9,10,11,12,20]. Content validity was evaluated by two specialists in paediatric dentistry, who independently assessed the relevance, clarity, and wording of each item; minor revisions were implemented according to their feedback. The revised questionnaire was pilot-tested on 15 students, whose responses were not included in the final analysis, to confirm clarity and completion time. Content validity was thus established qualitatively through expert review and pilot testing; a formal content validity index was not calculated.

The final instrument comprised 17 items organised into six domains: (1) sociodemographic characteristics (sex, age, year of study); (2) knowledge of child abuse and neglect; (3) knowledge of dental neglect; (4) attitudes toward the identification and reporting of suspected cases; (5) the perceived role of the dentist; and (6) interest in and preferred format of further education. Items used single-choice, multiple-response (“select all that apply”), and categorical attitude formats as appropriate.

### 2.5. Knowledge Recognition Score

An indicator-recognition score was derived from the four multiple-response (“select all that apply”) items addressing (i) recognised forms of child abuse, (ii) clinical indicators of physical abuse, (iii) indicators of dental neglect, and (iv) consequences of dental neglect. Within these four items, all listed options were genuine indicators, while a single “None of the above” option served as a distractor. Respondents received one point for each correct indicator selected; selection of the “None of the above” distractor contributed no points. Item scores were summed to produce a total recognition score with a theoretical range of 0 to 24, corresponding to the 24 correct indicators distributed across the four items (4, 7, 7, and 6 indicators, respectively). Because the four items did not include plausible incorrect options, this measure should be interpreted as reflecting the *breadth of recognition* of established indicators.

### 2.6. Reliability

The internal consistency of the recognition instrument was assessed using the Kuder–Richardson formula 20 (KR-20), the equivalent of Cronbach’s α for dichotomously scored items. One indicator, recognition of physical abuse as a form of maltreatment, was endorsed by all participants and therefore carried zero variance; it was excluded from the reliability computation. Across the remaining 23 dichotomous indicators, KR-20 was 0.73, indicating acceptable internal consistency.

### 2.7. Statistical Analysis

Data were exported from Microsoft Forms and analysed using Microsoft Excel (Microsoft Corp., Redmond, WA, USA) and IBM SPSS Statistics, version 27.0 (IBM Corp., Armonk, NY, USA). Categorical variables were summarised as frequencies and percentages, and the recognition score as mean ± standard deviation. Because the recognition score was a bounded count variable that was not normally distributed, as confirmed by the Kolmogorov–Smirnov test, between-group comparisons used the non-parametric Mann–Whitney U test, comparing scores by sex, by training stage (6th-year interns vs. 4th–5th-year clinical students), and by prior CAN education; effect sizes were expressed as the rank-biserial correlation. Differences in categorical attitudes by prior education were assessed with the χ^2^ or Fisher’s exact test, as appropriate. As a sensitivity analysis, all recognition-score comparisons were repeated after excluding three respondents who had selected every listed option, including the “None of the above” distractor, in the forms-of-abuse item; this did not materially change the results (overall mean 19.30 vs. 19.21) and did not alter the subsequent regression models.

To identify independent correlates while adjusting for potential confounding, a general linear model (multiple linear regression) was fitted with the total recognition score as the dependent variable and sex, stage of training, prior CAN education, and self-rated familiarity with dental neglect as predictors; results are reported as unstandardised coefficients (B) with 95% confidence intervals (CI), partial η^2^ effect sizes, and adjusted (marginal) means. As a sensitivity analysis, the model was refitted with heteroskedasticity-consistent (HC3) standard errors, which did not alter the conclusions. Two binary logistic regression models were then fitted with the intention to report a suspected case (yes vs. unsure/no) and endorsement of the dentist’s role in recognising CAN (yes vs. unsure/no) as dependent variables, and prior CAN education, sex, stage of training, and the recognition score as covariates; results are reported as adjusted odds ratios (aOR) with 95% CI. A two-sided *p*-value < 0.05 was considered statistically significant. The dichotomisation of intention to report (readiness for immediate reporting, “yes”, vs. “unsure”/”no”) was specified a priori to reflect clinically meaningful readiness to act; because “unsure” and “no” nevertheless represent distinct positions, intention to report was additionally modelled as an ordinal outcome (no < unsure < yes) using ordinal logistic regression as a sensitivity analysis, and the conclusions were unchanged. Because the recognition score reflects the breadth of endorsement of established indicators rather than validated knowledge, the multivariable models are regarded as exploratory and hypothesis-generating, and the resulting associations should not be interpreted as evidence of clinical competence or as validated predictive relationships.

## 3. Results

The questionnaire achieved a 57.5% response rate (*N* = 115). Among participants, 41 were male and 74 female; 50 were sixth-year students (interns) and 65 were in their clinical education (fourth–fifth year). Most students (*n* = 77) reported prior education on child abuse and neglect, primarily through university lectures, while 33% (*n* = 38) had not received any formal education on the subject. University lectures (58%) and online sources (25%) were listed as the main source of information about the topic, followed by scientific articles, seminars and clinical settings.

All participants (100%) recognized physical abuse as a form of child abuse, followed by sexual (97%) and emotional abuse (90%). A considerably smaller proportion (approximately 75%) identified neglect as a form of abuse. Only 3% of respondents did not consider any of the listed forms to constitute abuse (Table 1).

When asked which conditions might indicate the presence of physical abuse in a child, the majority of participants (87.8%) identified bruises or injuries of unclear origin as potential indicators. Inconsistent explanations for injuries (84.3%), recurrent trauma (82.6%), and atypical child behavior characterized by fear, anxiety, or excessive caution (82.6%) were also frequently recognized as warning signs. Jaw fractures (80%) and tooth fractures or avulsions (75.7%) were likewise regarded as significant clinical findings suggestive of abuse. A smaller proportion (62.6%) associated torn frenulum or mucosa with physical abuse, despite this being a well-documented indicator in the literature. None of the respondents selected the option indicating that none of the listed conditions were related to abuse (Table 2).

With respect to dental neglect, the majority (67%) indicated familiarity with the concept, but only 22% (*n* = 25) indicated that they were confident in the subject. The specific indicators of dental neglect and its consequences recognised by participants are presented in Table 3 and Table 4; the least frequently recognised items were frequent emergency dental visits (60.9%) and orofacial pain and infection (67.0%) among the neglect indicators, and poor school performance (52.2%) among the consequences. 66% of the respondents associated dental neglect with broader neglect patterns, and 78% (*n* = 90) acknowledged the dentist’s role in identifying abuse and neglect. 20% of the students were not certain whether the dentist had any role in identification of CAN. However, 36% of students reported that they were uncertain or unwilling to report suspected cases of child abuse and neglect to the appropriate authorities (Table 5).

To compare recognition scores between female and male participants, as well as between interns and clinical students, the Mann–Whitney U test was applied. Female participants demonstrated significantly higher recognition scores compared with male participants (*p* = 0.002). In contrast, no statistically significant difference was observed between the scores of interns and clinical (4th, 5th year) students, indicating that the year of study did not substantially influence recognition in this area (Table 6).

When asked about the main reasons for not reporting suspected cases, the most frequently cited factors were *fear of consequences for the child (75.7%)* and *unfamiliarity with reporting procedures (77.4%)*. These were followed by *fear of legal consequences (66%)* and *insufficient knowledge regarding the recognition of signs of abuse and neglect* (64.3%) (Table 7).

When questioned regarding the adequacy of the education they had received on the subject, a majority of students (51%) reported that it was insufficient, while 30% were uncertain. Only 18% considered the provided education to be adequate. Notably, 95% of respondents agreed that further education on the topic of CAN is warranted. Regarding the preferred format for such additional training, 71% favored practical seminars incorporating clinical cases, followed by lectures (56%) and online modules (42%) (Table 7).

**Recognition scores and attitudes by prior CAN education.** Overall recognition scores did not differ significantly between students who reported prior CAN education and those who did not (19.52 ± 2.92 vs. 18.84 ± 4.39; *p* = 0.713). At the component level, however, trained students recognized a significantly broader range of forms of abuse (3.86 vs. 3.24 of 4; *p* < 0.001; Cohen’s d = 0.97), whereas the physical-abuse and dental-neglect indicator sub-scores did not differ (all *p* > 0.6). In contrast, attitudes and reporting readiness differed markedly by prior education: trained students were more likely to endorse the dentist’s role in recognizing CAN (88.3% vs. 57.9%; *p* = 0.001) and to state that they would report a suspected case (66.2% vs. 39.5%; *p* = 0.011), and were somewhat more likely to consider their training adequate (23.4% vs. 7.9%; *p* = 0.078). Familiarity with dental neglect, the view that dental neglect signals general neglect, and interest in further education did not differ significantly (all *p* > 0.25) (Table 8).

**Independent correlates (multivariable analysis).** In the general linear model, female sex was the only independent predictor of a higher recognition score (B = 1.89; 95% CI 0.57–3.20; *p* = 0.005; partial η^2^ = 0.07; adjusted means 19.97 for female vs. 18.08 for male students), with self-rated familiarity showing a borderline association (B = 1.07; 95% CI −0.06–2.20; *p* = 0.064; partial η^2^ = 0.03); stage of training and prior education were not associated (Table 9). In the logistic models, prior CAN education and the recognition score were each independently associated with the intention to report (prior education aOR 3.31, 95% CI 1.30–8.47, *p* = 0.012; recognition score aOR 1.32 per point, 95% CI 1.14–1.53, *p* < 0.001) and with endorsement of the dentist’s role (prior education aOR 5.76, 95% CI 1.85–17.96, *p* = 0.003; recognition score aOR 1.42, 95% CI 1.19–1.70, *p* < 0.001); sex and stage of training were not independently associated with either outcome (Table 9).

## 4. Discussion

Child abuse and neglect remain a critical public health issue worldwide, with long-term consequences for children’s physical, psychological, and social development [21,22]. Among the various forms of maltreatment, dental neglect represents a frequently overlooked but clinically significant indicator of broader neglectful or abusive environments [23,24,25]. Because dental professionals are uniquely positioned to observe oral and maxillofacial signs of neglect, they play an essential role in the early identification and reporting of suspected cases. Routine examinations provide dentists with regular access to children, often revealing patterns of untreated caries, recurrent infections, trauma, and poor oral hygiene that may serve as warning signs of more systemic harm. Despite this strategic position, the international literature consistently highlights that many dental practitioners and students feel inadequately trained, insufficiently informed about reporting procedures, or afraid of potential legal and interpersonal repercussions [8]. For this reason, evaluating the knowledge, preparedness, and attitudes of dental students toward child abuse and neglect, including dental neglect, is crucial. Students represent the next generation of dental professionals; their level of awareness and confidence in recognizing and reporting maltreatment directly influences future clinical practice and child-safety outcomes. Understanding existing gaps in knowledge and identifying perceived barriers can guide curriculum redesign, professional training, and institutional policies aimed at strengthening the safeguarding role of dental practitioners.

Although two-thirds of participants (67%) reported prior education on CAN, primarily via university lectures, over half still perceived the education received as insufficient (51%), and almost all expressed a need for additional training (94.8%). This pattern aligns closely with international evidence that training exposure does not reliably translate into perceived readiness or procedural competence. For example, a national survey of Saudi dental graduates reported that while many had received education about CAN (73.3%), most were dissatisfied with the amount of CAN education (56.4%), only 39.5% felt confident identifying CAN, and only 9.7% knew where to report [18]. A German study comparing dental and medical students likewise found low satisfaction with CAN-related training and strikingly low preparedness: only 1.3% of dental students felt adequately prepared to deal with CAN, and only 7% of all participants felt able to report without help [10].

Compared with the near-universal recognition of physical abuse, neglect was less consistently identified in our cohort. This is clinically important because neglect, including dental neglect, may be more prevalent, more chronic, and more easily normalized or misattributed to socioeconomic hardship. Contemporary dental literature emphasizes that neglect requires careful contextual assessment, including distinguishing deliberate failure to seek care from barriers such as access limitations, finances, or parental capacity; these issues complicate recognition and reporting decisions [6,26].

Our data suggest moderate familiarity with the concept of dental neglect (67% “somewhat familiar”) but low confidence (only 21.7% “very well” familiar). This mirrors broader evidence that uncertainty is common even among qualified clinicians: in one UK-based survey, only a minority felt adequately trained in referral related to suspected child dental neglect, and barriers included uncertainty of diagnosis and lack of knowledge of referral pathways [27]. In our cohort, 66.1% linked dental neglect to general neglect patterns, which is consistent with the literature describing untreated oral disease as a potential marker of broader neglect, while also cautioning that not all poor oral health reflects willful caregiver omission [28,29].

A study in Turkey found that 58.6% of dental students believed that child abuse and neglect should be reported legally, but a similar proportion (60.9%) stated that they might not report suspected cases due to the possibility of making a misdiagnosis [13]. This is in accordance with our study, in which 57.4% of the students believed CAN cases should be reported; however, the most common barrier to not reporting in our study was lack of awareness of reporting procedures (77.4%), while insufficient training in recognising the signs of CAN was reported by 64.3% of the students. In Brazil, nearly half of dental students reported not knowing what measures to take in suspected cases, despite high self-reported awareness of child abuse [19]. In Saudi dental graduates, knowledge of *where* to report was particularly low (9.7%) [18]. These findings strongly support embedding clear, locally applicable reporting pathways into undergraduate training, including documentation standards, consultation routes, and multidisciplinary referral structures, rather than focusing solely on definitions and clinical signs. More broadly, a recent scoping review of dental health personnel synthesised the same core obstacles to reporting (diagnostic uncertainty, lack of knowledge, and fear of consequences), reinforcing that these barriers are consistent across settings and that targeted education and clearer referral pathways are needed to overcome them [30].

Female students in our sample achieved significantly higher recognition scores than males (*p* = 0.002). Similar patterns have been reported elsewhere; for example, in a large Saudi study, female participants had higher odds of adequate knowledge scores [18]. However, gender differences are not universal, and some studies report no significant gender effect [13], highlighting that gender likely acts as a proxy for other factors (educational engagement, prior exposure, risk perception) rather than as a direct determinant. These score differences should be interpreted with caution, as the instrument cannot distinguish accurate recognition from a general select-all response tendency, which may be present even among respondents who did not select the contradictory “None of the above” option.

Interestingly, our study found no significant difference between interns (6th year) and clinical (4th–5th year) students. This contrasts with studies reporting increasing competence with seniority [9,12,13,31]; only one other study also found no significant difference between year groups [20]. A plausible explanation in our setting is that CAN content is not progressively reinforced across the clinical years and that informal exposure in clinics is rare, variable, and not systematically linked to competency development.

Prior education showed a striking split. It was not associated with an increase in students’ overall recognition of maltreatment indicators; at the item level it was associated only with recognising the general forms of abuse, the content covered in lectures, and not with physical-abuse or dental-neglect signs. Yet students with prior education were far more ready to act: about three times more likely to say they would report a case and almost six times more likely to accept the dentist’s role. Because of the observational, cross-sectional design, these associations cannot be interpreted as causal effects of teaching; nonetheless, prior education was associated more strongly with attitudes and readiness to report than with clinical recognition, suggesting that knowing and being ready to act are not the same thing [9,10,11,12,13]. Teaching should therefore combine how to recognise the signs with clear, local rules on how and where to report. Moreover, because we measured self-reported intention rather than actual reporting behaviour, the well-documented intention-behaviour gap means that stated readiness may overestimate real-world reporting once these students enter practice.

Our findings align with recent evidence from other European settings. Among medical and nursing students in Greece, overall CAN knowledge was likewise unsatisfactory and neglect was recognised as a form of maltreatment by only a minority, although, as in our cohort, most students acknowledged their future safeguarding responsibility and wished for further training [16]. Among Lithuanian dental professionals, knowledge and attitudes were only moderate and varied across professional groups, with fear of negative consequences and professional uncertainty identified as key barriers to reporting [17]; these mirror the leading barriers in our sample, namely unfamiliarity with reporting procedures (77.4%) and fear of consequences for the child (75.7%). The higher recognition scores we observed among female students are also consistent with the Lithuanian data, in which female respondents demonstrated greater knowledge [17], and with earlier reports [18], supporting the view that sex may act as a proxy for educational engagement and risk perception rather than a direct determinant.

Our respondents overwhelmingly supported additional education and preferred practical seminars with clinical cases (71.3%). This preference matches what the broader evidence implies: students often learn *concepts* but remain uncertain about *process*, especially documentation, differential assessment (accidental versus non-accidental injury), multidisciplinary escalation, and legal and ethical responsibilities [10]. Accordingly, a structured educational package should include case-based, scenario-driven seminars; hands-on documentation workshops; clear reporting algorithms tailored to Bulgaria’s local safeguarding pathway (who to contact, how, and what to document); and interprofessional sessions involving pediatrics, social work and child protection, forensic medicine, and legal perspectives, reflecting recommendations that interdisciplinary approaches improve readiness. Recent evidence on educational interventions for health professionals similarly indicates that structured, practice-oriented and simulation-based training improves the recognition and reporting of maltreatment [32,33]. Coordinated, intersectoral collaboration between healthcare and child-protection services has likewise been identified as essential for an effective response to violence against children [34].

Several limitations should be acknowledged. First, the data are self-reported and may be subject to response or social-desirability bias, and actual reporting behaviour could not be assessed because participants were predominantly pre-clinical students; self-reported intention may therefore not fully translate into real-world reporting (the intention-behaviour gap). Second, higher scores of the recognition-score indicate broader recognition of established indicators rather than diagnostic competence in real clinical situations: because each item contained only a single distractor, it is susceptible to a select-all strategy, so the score and the observed sex difference should be interpreted cautiously; the use of “None of the above” as the only distractor is itself a recognised limitation in the assessment literature, where such items can affect item difficulty, discrimination, and respondents’ confidence [35,36]; the KR-20 coefficient indicates internal consistency only, not construct or discriminant validity, and content validity was established qualitatively (expert review and pilot testing) without a formal content validity index. Three respondents gave internally inconsistent answers, but a sensitivity analysis excluding them did not change the results. Third, as an observational cross-sectional study, the design precludes causal inference regarding prior education and attitudes or reporting intention. Finally, the study was conducted in a single institution with a 57.5% response rate and an anonymous design that precludes non-response analysis; recruitment also differed by training stage (a QR code in lectures for 4th- and 5th-year students versus institutional e-mail for interns), and curricula addressing CAN differ among Bulgarian faculties, all of which may introduce selection or mode bias and limit generalizability. Future research should focus on the longitudinal assessment of training interventions, comparison between institutions with different curricular approaches, and exploration of real-world reporting behaviors among practicing dentists. Strengthening the educational framework and operational protocols may ultimately enhance the role of dental professionals in safeguarding children.

## 5. Conclusions

This cross-sectional survey among clinical dental students in Plovdiv highlights important gaps between awareness of child abuse and neglect and self-reported readiness to report it. Within the limitations of the study, prior CAN education was associated with a greater intention to report and stronger endorsement of the dentist’s safeguarding role rather than with broader recognition of indicators. The findings support strengthening undergraduate education through structured, practice-oriented training and clear, locally applicable reporting pathways, so that future dentists are better prepared to protect vulnerable children.

## Figures and Tables

**Table 1:** Recognized forms of child abuse (*N* = 115).

Forms of Abuse	*n*	%
Physical abuse	115	100
Emotional abuse	104	90.4
Sexual abuse	112	97.4
Neglect	86	74.8
None of the above	3	2.6

**Table 2:** Conditions indicating possible physical abuse in a child (*N* = 115).

Condition/Indicator	*n*	%
Torn frenulum or mucosa	72	62.6
Bruises/injuries of unclear origin	101	87.8
Tooth fracture or avulsion	87	75.7
Jaw fracture	92	80.0
Recurrent injuries	95	82.6
Inconsistent explanations for injuries	97	84.3
Atypical child behavior (fearful, anxious, overly cautious)	95	82.6
None of the above	0	0.0
Other	3	2.6

**Table 3:** Recognition of dental neglect indicators (*N* = 115).

Indicator of Dental Neglect	*n*	%
Multiple untreated dental caries	109	94.8
Pain and infection in the orofacial region	77	67.0
Irregular preventive dental check-ups	88	76.5
Repeated failure to attend scheduled dental appointments	96	83.5
Frequent emergency dental visits	70	60.9
Poor oral hygiene	94	81.7
Untreated dental trauma	88	76.5
None of the above	0	0.0

**Table 4:** Recognition of the consequences of dental neglect (*N* = 115).

Consequence of Dental Neglect	*n*	%
Pain and infection	105	91.3
Orthodontic anomalies (malocclusion)	90	78.3
Difficulties with eating and speech	102	88.7
Delayed child development	84	73.0
Poor school performance	60	52.2
Low self-esteem	94	81.7
None of the above	0	0.0

**Table 5:** Participants’ training, awareness, and attitudes regarding child abuse and dental neglect (*N* = 115).

Question	Answer	*n*	%
Have you received training on recognizing indicators of child abuse and neglect (CAN)?	Yes	77	67.0
	No	38	33.0
If yes, where did you receive this training? (Select all that apply)	University lectures	67	58.3
	Was not trained	38	33.0
	Online sources	29	25.2
	Scientific articles	14	12.2
	Seminars and courses	6	5.2
	Clinical practice	4	3.5
	Forensic medicine curriculum	1	0.9
To what extent are you familiar with the term “dental neglect”?	Somewhat familiar	77	67.0
	Very familiar	25	21.7
	Poorly familiar	13	11.3
Do you think dental neglect is a sign of general child neglect (e.g., poor overall hygiene, malnutrition)?	Yes	76	66.1
	Not sure	27	23.5
	No	12	10.4
Do you think dentists have a role in recognizing child abuse and neglect?	Yes	90	78.3
	Not sure	23	20.0
	No	2	1.7
If you suspected a case of child abuse and neglect, would you report it to the authorities?	Yes	66	57.4
	Not sure	42	36.5
	No	7	6.1

**Table 6:** Comparison of indicator-recognition scores by sex and year of study (Mann–Whitney U test).

Characteristic	Category	*N* (%)	Mean Score (Mean ± SD)	*p*-Value
Sex	Male	41 (35.7)	17.90 ± 3.57	0.002
	Female	74 (64.3)	20.07 ± 3.19	
Year of study	Intern	50 (43.5)	18.92 ± 3.38	0.244
	Clinical (4th–5th year)	65 (56.5)	19.49 ± 3.48	
Total	—	115 (100)	19.30 ± 3.47	—

**Table 7:** Training adequacy, interest, and barriers to reporting child abuse and neglect (*N* = 115).

Question	Answer	*n*	%
Which of the following do you consider a barrier to reporting CAN cases? (Select all that apply)	Lack of awareness of reporting procedures	89	77.4
	Fear of consequences for the child	87	75.7
	Fear of legal consequences	76	66.1
	Lack of knowledge about recognizing the signs of CAN	74	64.3
	Fear of physical retaliation	60	52.2
Do you think the education you received about recognizing the signs of CAN is sufficient?	No	59	51.3
	Not sure	35	30.4
	Yes	21	18.3
Are you interested in further education about recognizing and reporting CAN?	Yes	109	94.8
	No	6	5.2
What type of education do you find most beneficial? (Select all that apply)	Practical seminars with case reports	82	71.3
	Lectures	64	55.7
	Online modules	48	41.7
	Not interested	2	1.7

**Table 8:** Knowledge and attitudes by prior CAN education (*N* = 115).

Variable	Received Education (*n* = 77)	Did Not Receive Education (*n* = 38)	*p*-Value
***Knowledge—mean ± SD***			
Total recognition score (0–24)	19.52 ± 2.92	18.84 ± 4.39	0.713
Recognised forms of abuse (0–4)	3.86 ± 0.45	3.24 ± 0.91	<0.001
Physical-abuse indicators (0–7)	5.56 ± 1.29	5.63 ± 1.36	0.674
Dental-neglect indicators (0–7)	5.44 ± 1.28	5.34 ± 1.46	0.781
Dental-neglect consequences (0–6)	4.66 ± 0.99	4.63 ± 1.34	0.832
***Attitudes/readiness—% responding “yes”***			
Endorses the dentist’s role in recognising CAN	88.3%	57.9%	0.001
Would report a suspected case	66.2%	39.5%	0.011
Dental neglect signals general neglect	70.1%	57.9%	0.274
Very familiar with dental neglect	24.7%	15.8%	0.397
Considers own training sufficient	23.4%	7.9%	0.078
Interested in further education	96.1%	92.1%	0.395

Continuous variables compared with the Mann–Whitney U test; categorical variables with the χ^2^ or Fisher’s exact test. CAN, child abuse and neglect.

**Table 9:** Multivariable correlates of recognition score, intention to report, and endorsement of the dentist’s role (*N* = 115).

Predictor	B/aOR	95% CI	*p*-Value
***Model 1. Linear regression—total recognition score (B; R^2^ = 0.12)***			
Female sex (ref. male)	1.89	0.57 to 3.20	0.005
Intern (ref. 4th–5th-year clinical)	−0.54	−1.81 to 0.72	0.398
Prior CAN education (ref. none)	0.14	−1.22 to 1.50	0.838
Familiarity with dental neglect	1.07	−0.06 to 2.20	0.064
***Model 2. Logistic regression—intention to report (aOR)***			
Prior CAN education (ref. none)	3.31	1.30 to 8.47	0.012
Recognition score (per point)	1.32	1.14 to 1.53	<0.001
Female sex (ref. male)	1.55	0.63 to 3.84	0.343
Intern (ref. 4th–5th-year clinical)	0.73	0.30 to 1.74	0.472
***Model 3. Logistic regression—endorses dentist’s role (aOR)***			
Prior CAN education (ref. none)	5.76	1.85 to 17.96	0.003
Recognition score (per point)	1.42	1.19 to 1.70	<0.001
Female sex (ref. male)	1.68	0.55 to 5.16	0.362
Intern (ref. 4th–5th-year clinical)	0.97	0.31 to 3.03	0.959

B, unstandardised linear-regression coefficient; aOR, adjusted odds ratio; CI, confidence interval. Reference categories: male sex, 4th–5th-year clinical student, no prior CAN education. The linear-model association for female sex was confirmed with HC3 robust standard errors (*p* = 0.008).

## Data Availability

The data presented in this study are available on request from the corresponding author. The data are not publicly available as they form part of an ongoing intra-university research project and given the sensitive nature of the topic.

## Abbreviations

The following abbreviations are used in this manuscript:

CAN	Child abuse and neglect
